# Spatial transcriptomics at the brain-electrode interface in rat motor cortex and the relationship to recording quality

**DOI:** 10.1088/1741-2552/ad5936

**Published:** 2024-07-31

**Authors:** Quentin Whitsitt, Akash Saxena, Bella Patel, Blake M Evans, Bradley Hunt, Erin K Purcell

**Affiliations:** 1 Department of Biomedical Engineering and Institute of Quantitative Health Science and Engineering, Michigan State University, East Lansing, MI 48824, United States of America; 2 Department of Electrical and Computer Engineering, Michigan State University, East Lansing, MI 48824, United States of America

**Keywords:** spatial transcriptomics, microelectrode implants, foreign body reaction, differential gene expression, electrophysiology

## Abstract

Study of the foreign body reaction to implanted electrodes in the brain is an important area of research for the future development of neuroprostheses and experimental electrophysiology. After electrode implantation in the brain, microglial activation, reactive astrogliosis, and neuronal cell death create an environment immediately surrounding the electrode that is significantly altered from its homeostatic state. *Objective.* To uncover physiological changes potentially affecting device function and longevity, spatial transcriptomics (ST) was implemented to identify changes in gene expression driven by electrode implantation and compare this differential gene expression to traditional metrics of glial reactivity, neuronal loss, and electrophysiological recording quality. *Approach.* For these experiments, rats were chronically implanted with functional Michigan-style microelectrode arrays, from which electrophysiological recordings (multi-unit activity, local field potential) were taken over a six-week time course. Brain tissue cryosections surrounding each electrode were then mounted for ST processing. The tissue was immunolabeled for neurons and astrocytes, which provided both a spatial reference for ST and a quantitative measure of glial fibrillary acidic protein and neuronal nuclei immunolabeling surrounding each implant. *Main results*. Results from rat motor cortex within 300 *µ*m of the implanted electrodes at 24 h, 1 week, and 6 weeks post-implantation showed up to 553 significantly differentially expressed (DE) genes between implanted and non-implanted tissue sections. Regression on the significant DE genes identified the 6–7 genes that had the strongest relationship to histological and electrophysiological metrics, revealing potential candidate biomarkers of recording quality and the tissue response to implanted electrodes. *Significance*. Our analysis has shed new light onto the potential mechanisms involved in the tissue response to implanted electrodes while generating hypotheses regarding potential biomarkers related to recorded signal quality. A new approach has been developed to understand the tissue response to electrodes implanted in the brain using genes identified through transcriptomics, and to screen those results for potential relationships with functional outcomes.

## 1. Introduction

Intracortical microelectrode implants have the capacity to stimulate nervous tissue and/or record electrical signals from the brain. This functionality allows for a wide range of applications, from treating debilitating neurological diseases to preclinical and basic neuroscientific research. Clinical applications of these devices have progressed rapidly over the past decade and have recently gained newfound interest due to the potential for use in brain–computer interfaces (BCIs). Recent advances in recording-based implants have restored quadriplegic [1, 2] or quadriparesis [3] patients’ ability to communicate. BCI research has shown the capability of recording implants in motor cortex to drive movement of a robotic limb or computer cursor [4, 5]. Generally, intracortical electrodes are used in these applications to record electrical signals from specific areas of the brain. An algorithm is then trained to decode the electrical signals into meaningful information that can be used to drive the BCI and produce the desired outcome.

Limitations to the long-term use of recording implants *in vivo* include observations of a decrease in signal quality over time [6–10] as well as signal instability [6, 10–12]. Decline of signal quality is characterized by decreased amplitude of unit waveforms, number of resolvable units, signal-to-noise ratio, and an increase in impedance. All these trends can contribute to a decline in device function by reducing information transfer at the brain-electrode interface [13]. This creates limitations for current BCIs because loss of information directly affects decoder performance [13], and it also creates limitations in research settings when trying to study complex and precise neurological activity over a long period of time.

There are multiple possible mechanisms that may lead to signal quality and stability decline [7, 14]. In addition to mechanical/electrical failure and micromotion, a long-standing line of inquiry into the origins of signal decay focuses on the biological response to implants in the brain, known as the foreign body reaction (FBR) [15, 16]. It has been predicted that ∼85% of firing rate variability, which can lead to decreased BCI performance, is due to physiological changes [8]. In the brain, the FBR is often characterized by a loss of neuronal density surrounding the implant, which has been shown in one report to decrease by 40% within 100 *µ*m [17]. Another prominent effect is the presence of reactive astrocytes surrounding the implant after 1 week post-implantation, shown by increased glial fibrillary acidic protein (GFAP) [15]. It has been shown that increased glial encapsulation can lead to increased impedance; however, it is not clear how increased impedance influences recording quality over time [9]. Furthermore, even though the FBR traditionally has been studied using the immunohistochemical (IHC) methods mentioned, neuronal loss and GFAP intensity do not necessarily predict signal stability [18]. Thus, the relationship between recording quality and the biological response remains unclear, and predictive biomarkers of signal quality have yet to be identified.

Transcriptomics has been a successful approach for identifying important biomarkers associated with diseases of the central nervous system (CNS). For example, transcriptomics has revealed a novel target, neddylation, that ameliorates the severity of multiple sclerosis in a murine disease model [19]. Transcriptomics has also been applied in Alzheimer’s disease (AD) research to expose genetic, cell-type specific regulators of myelination that are perturbed in AD, as well as sex differences in the cellular response to AD [20]. Additionally, a transcriptomics method which allows for gene expression to be spatially resolved has been applied in AD research to reveal groups of genes that are differentially expressed (DE) surrounding amyloid plaques [21]. Recently, a new focus in the study of the brain-electrode interface is the use of transcriptomics to study the FBR. Recent studies from multiple labs have illuminated specific genes that are DE around the implant compared to non-implanted brain tissue [22–24]. In an initial publication in this area, laser capture microscopy was used to dissect fixed tissue from near the device tract (<100 *µ*m), from tissue 500 *µ*m away from the device tract, and from a non-implanted animal. Following bulk RNA sequencing of the excised tissue samples, DE analysis revealed 157 differentially expressed genes (DEGs) at 24 h, 62 DEGs at 1 week, and 26 DEGs at 6 weeks post-implantation in comparison to non-implanted tissue. Differential expression DE analysis revealed several genes that were significantly DE between tissue 500 *µ*m from the device and naïve tissue, suggesting the spatial extent of differential gene expression can extend for several hundred microns from the device surface.

Here, in an extension of previous work [22], we report the application of a newer spatial transcriptomics (ST) method which has three advantages over our previous approach: (1) it reports the transcriptional profile of individual genes with improved, near cellular-scale resolution across the entire landscape of a tissue section containing the device, (2) it improves RNA quality compared to previous work through the use of fresh-frozen tissue samples, and (3) it allows quantitative immunohistochemistry and ST to be performed in the same tissue section, which allows gene expression data to be directly compared to traditional IHC data. We applied this technique to the brain-electrode interface in rats implanted with single shank, silicon microelectrode arrays implanted in motor cortex. All timepoints (24 h, 1 week, and 6 week implants) yielded significant, DE genes when comparing either implanted tissue sections to non-implanted tissue sections or within-section regions. Each timepoint revealed novel genes that have not been identified previously, as well as the spatial extent of each gene. We also provide an initial investigation into the relationship between gene expression within the electrode site and measurements of both recording quality (signal to noise ratio, amplitudes of multi-unit activity and local field potential (LFP) and traditional histological metrics (neuronal density and GFAP intensity). Using linear regression and principal component regression (PCR), predictive quality of individual and sets of significantly and DE genes were calculated for each metric. The predictive quality and the fold change of the genes were used to identify a small subset of genes which maximise both the parameters. These genes may deserve added attention as potential biomarkers for electrode-tissue integration which are based on functional outcomes.

## 2. Material and methods

### 2.1. Surgery, Implantation & Sample Preparation

All electrodes used for this study were single-shank, silicon, Michigan-style microelectrodes (A1*x*16–3 mm-100–703-CMLP, 15 *µ*m thickness, NeuroNexus Inc, Ann Arbor, MI). Adult, male, Sprague Dawley rats received implants in motor cortex using coordinates and procedures as previously described [22, 25]. Briefly, electrodes were inserted (+3.0 mm AP, 2.5 mm ML from Bregma, 2.0 mm deep) during isoflurane anaesthesia, and the surgical site was closed via a dental acrylic headcap. Post-operative analgesia was achieved with injected meloxicam (2 mg kg^−1^) and topical application of bupivacaine. At the appropriate timepoint (24 h, 1 week, or 6 weeks), animals were euthanized via sodium pentobarbital administration and decapitation, and brains were rapidly removed and cryo-embedded using liquid nitrogen (*n*= 3 rats/time point). Aligning with other studies using this ST method [21, 26, 27], animals were not perfused prior to euthanasia. Tissue was then cryosectioned from primary motor cortex at a depth of ∼1000 *µ*m and mounted on a Visium Spatial Gene Expression slide (10*x* Genomics, Pleasanton CA). All animal procedures were approved by the Michigan State University Animal Care and Use Committee.

### 2.2. Immunohistochemistry

Immediately prior to IHC, the tissue was fixed in cold methanol for 30 min. It was then blocked in a bovine serum albumin solution before being labelled using a neuronal nuclei (NeuN) primary antibody (Rb pAB to NeuN, Abcam, Cambridge, MA, Cat #:104225) at a concentration of 1:100 and a GFAP primary antibody (Monoclonal Anti-GFAP) antibody, Millipore Sigma, St. Louis, MO, Cat #: G3893-100) at a concentration of 1:400. Secondary antibodies used for staining were AlexaFluor 488 (Anti-rabbit IgG, Invitrogen, Eugene OR, Cat #: A11034) for conjugation to the NeuN primary and AlexaFluor 647 (Anti-mouse IgG, Invitrogen, Eugene, OR) for conjugation to the GFAP primary. Additionally, Hoechst (Life Technologies Corp. Eugene, OR) was used as a universal nuclei stain at a concentration of 1:1000. After staining, a Nikon A1R confocal microscope (Nikon, Tokyo, Japan) with a motorized stage was used to capture individual 20*x* magnification image tiles of each capture area, outlined with a fluorescent fiducial frame, on the Visium slide. The final wide-field image was stitched together from the individual image tiles by automated Nikon software.

Quantitative IHC was performed using a custom MATLAB script that has been previously described [25, 28–30]. Briefly, neuronal density was measured in 100 *µ*m bins radiating outwards from the surface of the tissue surrounding the electrode tract in implanted tissue and from a comparable area of tissue in naïve controls. This comparable area used from naïve controls was determined by relative location of larger brain structures such as distance from the glia limitans and stratal patterns of neuron density in the transverse sections of brain tissue. A similar strategy was used to estimate relative GFAP protein expression within each tissue sample with 10 *µ*m bins. Fluorescence intensity of GFAP staining was used as the proxy for amount of GFAP protein expression in this quantitative IHC method. A linear mixed model ANOVA with a Bonferroni post-hoc test was used to test the statistical significance of quantitative immunohistochemistry results (SPSS software, IBM), similarly to previously described work [22]. Statistical significance was defined at the *P* ⩽ 0.05 level.

### 2.3. Tissue permeabilization & complementary DNA (cDNA) synthesis

After imaging, tissue was enzymatically permeabilized for 18 min. This permeabilization time was established through an optimization experiment, where fluorescent cDNA was created on the slide to reveal the permeabilization time that maximized RNA release from a tissue section (supplementary figure 1). Although fluorescence intensity appears similar between 18 and 24 min trials, the 24 min trial showed evidence of over-permeabilization due to undefined cell body borders. After permeabilization and mRNA capture, reverse transcription was performed on the slide to transfer bound mRNAs’ nucleotide sequences to the capture oligos on the slide. The original mRNA strands were then released from the capture oligos and through a series of template switching and second strand synthesis steps, Final cDNA samples were produced which contained sequences of the originally bound mRNA molecules, unique molecular identifiers, and spatial barcodes. cDNA was then released from the slide via denaturation and transferred to a DNA/RNA LoBind microcentrifuge tube (Eppendorf, Cat #: 022431021). The cDNA from each capture area was quantified using qPCR and amplified using the number of cycles from qPCR required to achieve 25% of the peak fluorescence value. After amplification, the cDNA was purified using SPRIselect (Beckman Coulter Inc, Brea, CA), a paramagnetic bead-based size selection reagent.

To find the RNA Integrity Number (RIN) of the tissue, a ‘QIAshredder’ kit (Qiagen, Hilden, Germany) was used to homogenize collected cryosectioned samples. RNA was isolated using a RNeasy Mini Kit and initially assessed using the Qubit assay (Invitrogen, Waltham, MA). RIN was calculated at the MSU Genomics Core using the High Sensitivity RNA ScreenTape Assay on an Agilent 4200 TapeStation.

### 2.4. RNA sequencing and differential expression analysis

Samples were then transferred to the University of Michigan Advanced Genomics core for library preparation and sequencing. cDNA quality was assessed using the Tapestation 2200 (Agilent) and subjected to library preparation following the manufacturer’s protocol (10*x* Genomics). Final library quality was assessed using the LabChip GX (PerkinElmer). Pooled libraries were subjected to paired-end sequencing according to the manufacturer’s protocol (Illumina NovaSeq 6000). Bcl2fastq2 Conversion Software (Illumina) was used to generate de-multiplexed Fastq files and the SpaceRanger Pipeline (version: 2.0.0, 10*x* Genomics) was used to align reads and generate count matrices. Individual, spatially barcoded spots were excluded from SpaceRanger alignment based on a couple criteria. Some regions of tissue exhibited artifacts from cryosectioning such as folds, tears, and bubbles as well as artifacts from staining in the form of specks of high fluorescence intensity. Areas affected by both types of artifacts were removed from ST analysis. The main output of SpaceRanger is the ‘cloupe’ file, readable by ‘Loupe Browser’ software (version: 6.2.0, 10*x* Genomics), which was used for the following differential expression analysis. Raw fastq files were also analysed using FastQC (version: 0.11.7-Java-1.8.0_162) to verify sequencing quality.

Data was imported into Loupe Browser, where clusters of spots were selected to do a basic count measurement for each gene of interest as well as overall DE analysis. For distance measurements, images were binned using the open-source Fiji image analysis software [31] (version 2.6.0) to identify areas of tissue extending from the electrode tracts in 100 *µ*m radius increments. Spots were then clustered based on the bin their centroid was located in. Thus, the distance measurements represent a binning of the spots based on their centroid, not their full area.

Two methods of analysis were applied using Loupe Browser. The first measured the median normalized average (MNA) counts of genes of interest within each bin, starting at 0–100 *µ*m and extending out to a bin 900–1000 *µ*m from the electrode tract. The MNA counts for each gene in each bin were calculated through the Loupe Browser (6.2.0, 10x Genomics). The second method used was standard DE analysis, also calculated through Loupe Browser algorithms, between two clusters. The clusters chosen for comparisons included spots whose centroids were in the first three bins (0–300 *µ*m) at each timepoint, compared to the same area in naïve tissue, giving three overall comparisons. Distance measurements for naïve tissue were selected based on a similar strategy as quantitative IHC, using areas of naïve tissue comparable to the implanted tissue for analysis. ‘Low-count’ genes were excluded from analysis which removed genes with less than one count per spot, on average, in both clusters used for the DE comparison. DE genes were further filtered to reveal ‘significant’ DE genes which we define as genes with a *p*-value less than 0.05 and a log_2_(fold change) (LFC) ⩾0.6 or ⩽−0.6.

DEGs are then used in gene ontology (GO) analysis, which inputs lists of DEGs and produces key terms that describe the active biological processes. The Gene Ontology Enrichment Analysis and Visualization Tool (GOrilla [32]: https://cbl-gorilla.cs.technion.ac.il/) is used for this purpose, and significant (FDR < 0.05) and highly enriched GO terms are reported for each timepoint vs. naïve comparison.

### 2.5. Electrophysiology

Electrophysiological recordings were taken using an RZ2 BioAmp Processor (Tucker-Davis Technologies, Alachua, FL) from the implanted, 16-channel, Michigan-style electrodes using methods previously described [25]. Briefly, wideband data was sampled at ∼48 kHz in lightly isoflurane-anesthetized (∼1%) rats placed in a Faraday cage. Data were analysed offline in Matlab as described [33]. Recordings were taken at 24 h post-implantation, 1 week post-implantation, and then weekly until the terminal recording immediately prior to euthanasia. The metrics of interest to be extracted from these recordings are the average amplitudes of local field potential LFP, multiunit activity (MUA) and signal to noise ratio (SNR) averaged across all the 16 channels of the implant. SNR and MUA were calculated following the algorithm mentioned in the following paper [34] with a few modifications. In brief, the common average reference (CAR) was calculated and subtracted from each of the recordings to mitigate the effect of correlated noise sources [34]. Next, a band pass filter for 500–6000 Hz was applied on the CAR subtracted signals. For every sample outside the bounds of ±3.5 times standard deviations (STD), a 2.4 ms window centred at the absolute minimum of the signal was extracted and stored; the remaining data is noise. If the RMS of the noise floor was between 0.3 and 2 times the average RMS of the noise floor across all 16 sites on the array, the data from those channels was included in further analysis, removed if not (this serves as a method to detect and exclude damaged sites) [34]. To calculate the SNR, peak to peak (P2P) value of each the stored snippets was calculated, then averaged and divided by 6 times the standard deviation of the data comprising the noise floor. The SNR values obtained for each channel is further averaged across the channels to obtain an average SNR metric for implant. To calculate MUA, mean of the positive deflections (samples > 3.5 * STD) and the negative deflections (samples < −3.5 * STD) was calculated, and the P2P value of these 2 averages was the mean MUA amplitude for the electrode site. The mean MUA amplitude values for each site were averaged across all the channels to calculate the average MUA amplitude for that implant. To calculate the LFP amplitude, the raw data from every channel was passed through a 60 Hz notch filter to remove residual 60 Hz noise followed by application of a band pass filter with cut-offs at 1 Hz and 300 Hz. The amplitude for every channel was calculated as 6 times the standard deviation of the filtered data.

### 2.6. Identification of potential biomarkers of recording quality

All sequencing data is first normalized to its sequencing depth using Space Ranger, and the MNA is applied to the region of interest to normalize the mRNA reads for spots in that region. MNA is applied by multiplying every spot in the selected cluster of spots by the ratio of the total counts of that spot and the median of the total UMI counts of that cluster. As an additional quality assessment, each sample was inspected for non-linearity by assessing the relationship between the raw counts of GAPDH [35, 36] and *Actin-B* [35, 36] (commonly used housekeeping genes) to the total counts with respect to every spot. As housekeeping genes are involved in cellular maintenance, they are expected to be consistently expressed across the tissue (i.e. the raw counts should increase or decrease along with the total number of UMI counts at that spot). Our samples exhibited a linear relationship between total UMI counts and housekeeping genes (supplementary figures 2–9).

Following normalization and inspection of housekeeping gene expression, we first inspected the data via principal component analysis (PCA) applied to the MNA for the entire tissue section for every gene. PCA was also performed for the cluster of spots within 300 *µ*m of the electrode. This was done because PCA for the entire tissue extends beyond the boundaries of M1, and includes different regions, which could influence the results. For the naïve animals (*n* = 3), 3 non overlapping random electrode sites with a radius of 300 *µ*m were chosen for each animal to be included in the PCA analysis (supplementary figure 10). Individual animals’ results were then plotted against the calculated PC1 and PC2 to observe the relative variability in gene expression across different animals and time points.

To further explore the relationship between the genes and the metrics of interest (MUA, LFP, SNR, GFAP and ND), an elementary pipeline was established. Differential expression analysis and assessments of LFC is a standard approach to identify genes of interest [23, 37, 38]. In this study, we included an additional step of screening those genes with a potential relationship with the device presence for those that may also have a relationship with recording quality. Linear regression is a natural first step for assessing the strength of a relationship between two variables [39–42], and PCR is used when the number of independent variables outnumber the dependent variables [43, 44]. This is because PCR reduces a large set of correlated predictor variables to a smaller, less correlated set, called principal components, that still contains most of the information in the larger set. In addition to dimensionality reduction, it serves as a useful tool for exploration of data. Thus, our initial analysis considered 2 metrics, (1) a metric to reflect the gene’s possible participation in the tissue-electrode interaction: log fold change, and (2) a metric to reflect the strength of association between the 2 variables, for which we use the *R* ^2^ statistic of regression (either linear regression for 1 gene or PCR for multiple genes). These 2 metrics are used to select genes of interest and understand the relationship between the response and dependent variables.

First, we consolidated the list of significantly DE genes at the 24 h (553), 1 week (282) and 6 week (25) time points into one list of 645 total genes (excluding overlap). For the genes that were common across more than 1 timepoints, the LFC with the highest value was chosen to represent the LFC of that gene. We next sought to quantitatively evaluate the predictive quality of each gene and sets of genes on recording quality and IHC metrics using linear regression and PCR . Linear regression was performed on the electrode site MNA values for each individual gene, giving the *R* ^2^ statistic and the *p*-value (non-adjusted) of the F–statistic for each gene for each individual test. PCA analysis was performed on sets of genes in an iterative manner to obtain the coefficients along the PCs for every possible combination in that set. Linear regression analysis was performed between the obtained coefficients from PC1 of each set and metrics of recording quality and tissue response. This analysis produced an *R* ^2^ statistic and the non-adjusted *p*-value of the F–statistic for regression (PC1 obtained from each set of genes) to assess the strength and significance of the relationship between the expression of every gene and the MUA amplitude, LFP amplitude, GFAP intensity, and neuronal density for that tissue sample. The latter two metrics were calculated for the tissue within 300 *µ*m of the device interface, which captures the recordable radius of the device [45, 46]. To choose the most significant genes, LFC and average LFCs in case of sets of genes are plotted against the *R* ^2^ statistic to select the top 4–7 genes/gene sets with the combination of highest *R* ^2^ statistic (reflecting a possible relationship with histological or recording quality metrics) and the highest LFC/average LFC (capturing the genes that are most strongly affected by the device presence). The results of this pipeline for sets genes are available in the supplementary section (supplementary figure 11). The chosen genes and sets of genes are fitted onto the response variable using the intercept and the coefficient produced by the linear regression. In this way, we were able to visualize the relationships between individual genes, recording quality, and histology metrics, revealing potential candidate biomarkers associated with performance metrics and tissue response outcomes.

## 3. Results

### 3.1. Quantification of IHC markers (NeuN, GFAP) and recording quality associated with devices

**Figure 1.**
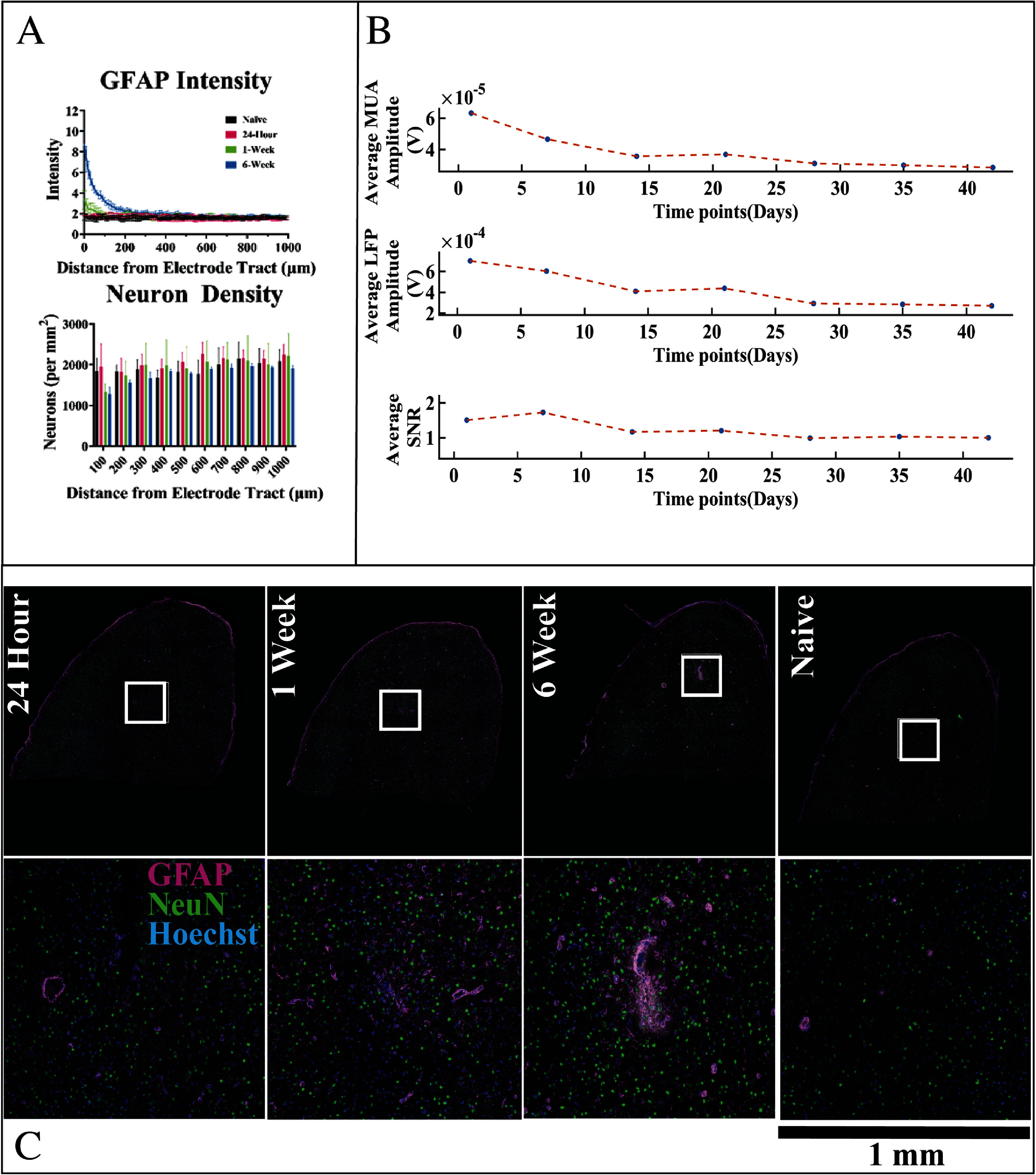
Tissue surrounding implanted electrodes displays significant gliosis and a progressive loss of recording quality over time. (A) The GFAP intensity within 100 *µ*ms of the device trended higher at 7 d post-implantation (*p* < 0.1) and is statistically significantly increased at 6 weeks post-implant in comparison to earlier time points (*p* < 0.001). Neuronal density was relatively reduced within 100 *µ*ms of the device at the 1 and 6 week time points, but the result did not reach statistical significance. (B) The average SNR, MUA and LFP amplitudes show a progressive decline in signal quality over the six-week time course of the study (*n* = 2–3 animals per time point) (C) Representative image of the IHC tissue at different time points (top row) alongside magnified images (bottom row) corresponding to region outlined by the white box. Transcriptomics and IHC data were taken from the same tissue slice for each rat.

**Figure 2.**
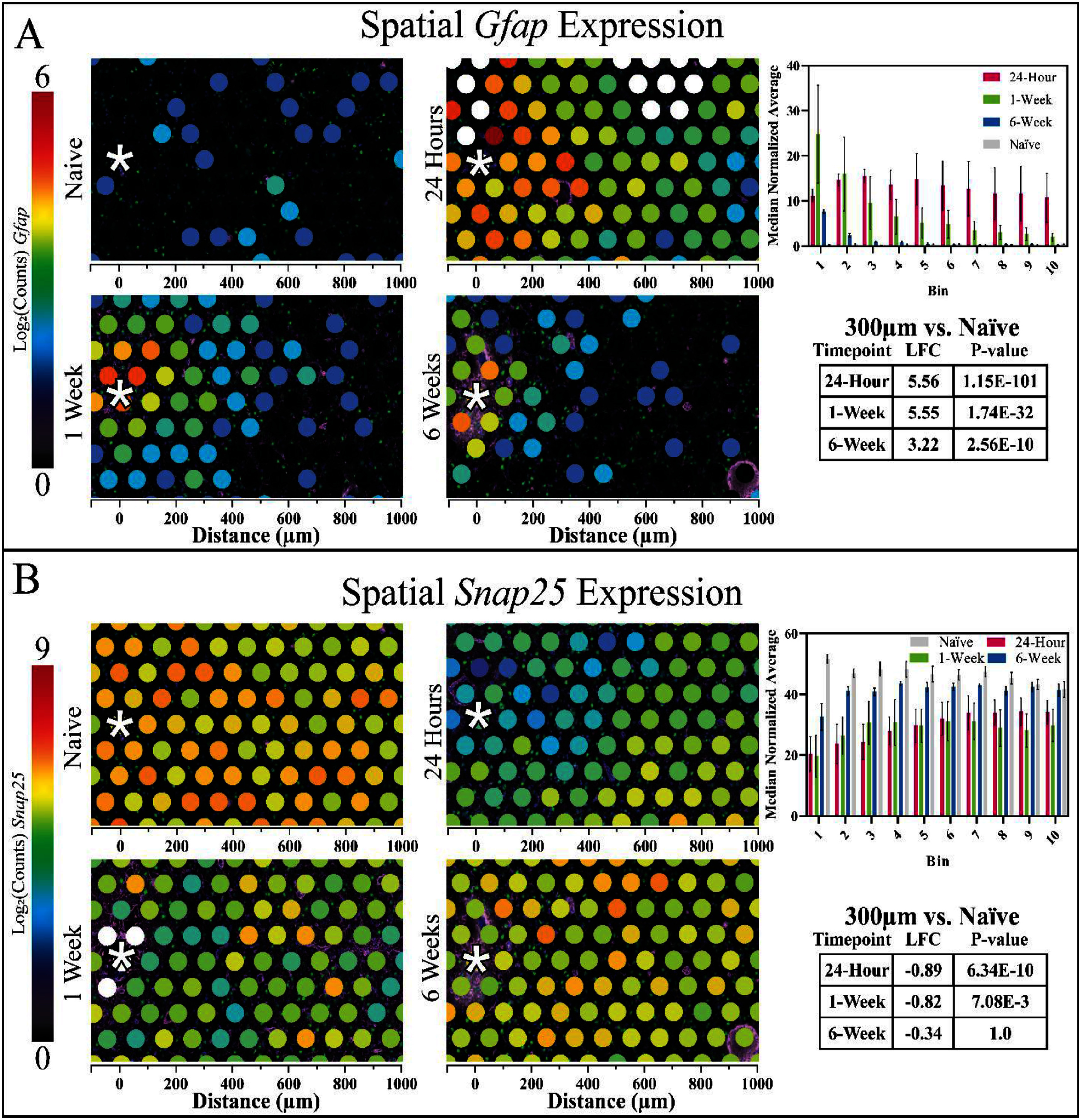
Gene expression data for *Gfap* (A) and *Snap25* (B) reinforce astroglial reactivity and neuronal loss following device implantation. The colour at each spot expresses the log_2_ (normalized counts) as a measure of the amount of gene expression, and the *x*-axis represents the distance from electrode site. The MNA of counts for each gene within the 100 *µ*m bins is centred at the neural implant site up to 1000 *µ*m. *Gfap* expression was significantly upregulated within 300 *µ*m of the implant at all time points, and *Snap25* expression was significantly downregulated within the same region at the 24 h and 1 week time points.

We first characterized the tissue response to electrodes using traditional histological methods (NeuN, GFAP quantification) and assessed the loss of signal quality over time. Quantitative immunohistochemistry revealed typical patterns of both reactive astrogliosis and neuronal loss surrounding the electrode implants (figure 1). Likewise, we noted a progressive decline in metrics of signal amplitude, similar to previous results obtained from Michigan-style silicon arrays implanted in rats. GFAP staining intensity, a commonly used biomarker to estimate astrocyte reactivity, shows a trend toward increased expression at 1 week (*p* < 0.1), and displays a statistically significant increase from naïve tissue at 6 weeks post-implantation within 100 *µ*m of the device interface (*p* < 0.001). Neuronal density, measured by the number of NeuN per area, trends lower at 1 week and 6 weeks post-implantation in the same region, but the result did not reach statistical significance (*p* > 0.05), likely due to the relatively limited number of samples probed per time point. ST yields a high quantity of information per sample for the expression of thousands of genes, but typically, relatively few samples are assessed due to associated costs [21, 47–49]. However, evidence of neuronal damage/loss is supported by a reduction in the expression of the neuron-specific *Snap25* gene (figure 2). The results align with previous descriptions of the tissue response to silicon electrodes reported in literature [50], and the complete set of twelve IHC samples are supplied as supplementary figures (supplementary figures 12 and 13). Based on a study using Nissl staining of rat motor cortex [51], implants and the tissue surrounding the electrode tract appear to be consistent with placement in layer 5 of the motor cortex, which was the target of this study due to the presence of large pyramidal neurons [52] and relevance to clinical applications [53–55].

### 3.2. Overview of gene expression results: quality and comparison with protein expression

Spatial distribution of UMI counts in each sample were mostly uniform (supplementary figure 14). UMI counts are equivalent to the total number of counts of all genes generated through sequencing. The number of UMI counts are used to verify adequate sequencing depth, which was consistent with the recommended range for all samples in this study on average (>50 000 UMI counts per spot). The bulk RIN scores for two separate samples tested with the tissue preparation method >7, which is improved over previous transcriptomics techniques reported post-fixation [22]. As a first analysis step, we benchmarked gene expression (*Gfap* and *Snap25*) to the IHC results (figure 2). *Gfap* gene expression and GFAP protein expression followed similar patterns, with an increase near the device at 1 and 6 weeks post- implantation; however, there were differences between *Gfap*/GFAP expression. At 24 h post-implantation, *Gfap* gene expression is elevated above naïve samples in all bins (1–10) (figure 2), which corresponds to distances 0–1000 *µ*m from the device tract. Gene and protein expression often do not display a one-to-one correspondence [56].

Additionally, while GFAP protein expression peaks at the interface 6 weeks post-implantation, ST found *Gfap* gene expression peaked at the interface 1 week post-implantation. Due to low *Rbfox3* expression (encodes NeuN protein), *Snap25* was used as a substitute neuronal marker for transcriptomic analysis [56]. In a report detailing the genetic profiles of three main types of brain cells (astrocytes, oligodendrocytes, and neurons), *Snap25* was the most highly enriched of the neuron-specific genes identified [57]. A subsequent report confirmed the neuronal specificity of *Snap25* and its exclusion from microglial expression [58].”

A decrease in *Snap25* expression was observed at all timepoints compared to naïve tissue. Comparing spots with centroids within 300 *µ*m of the device tract to the same area of spots in naïve tissue sections revealed 553 DE genes at 24 h, 282 DE genes at 1 week, and 25 DE genes at 6 weeks.

### 3.3. Differential gene expression between implanted and naïve tissue

**Figure 3.**
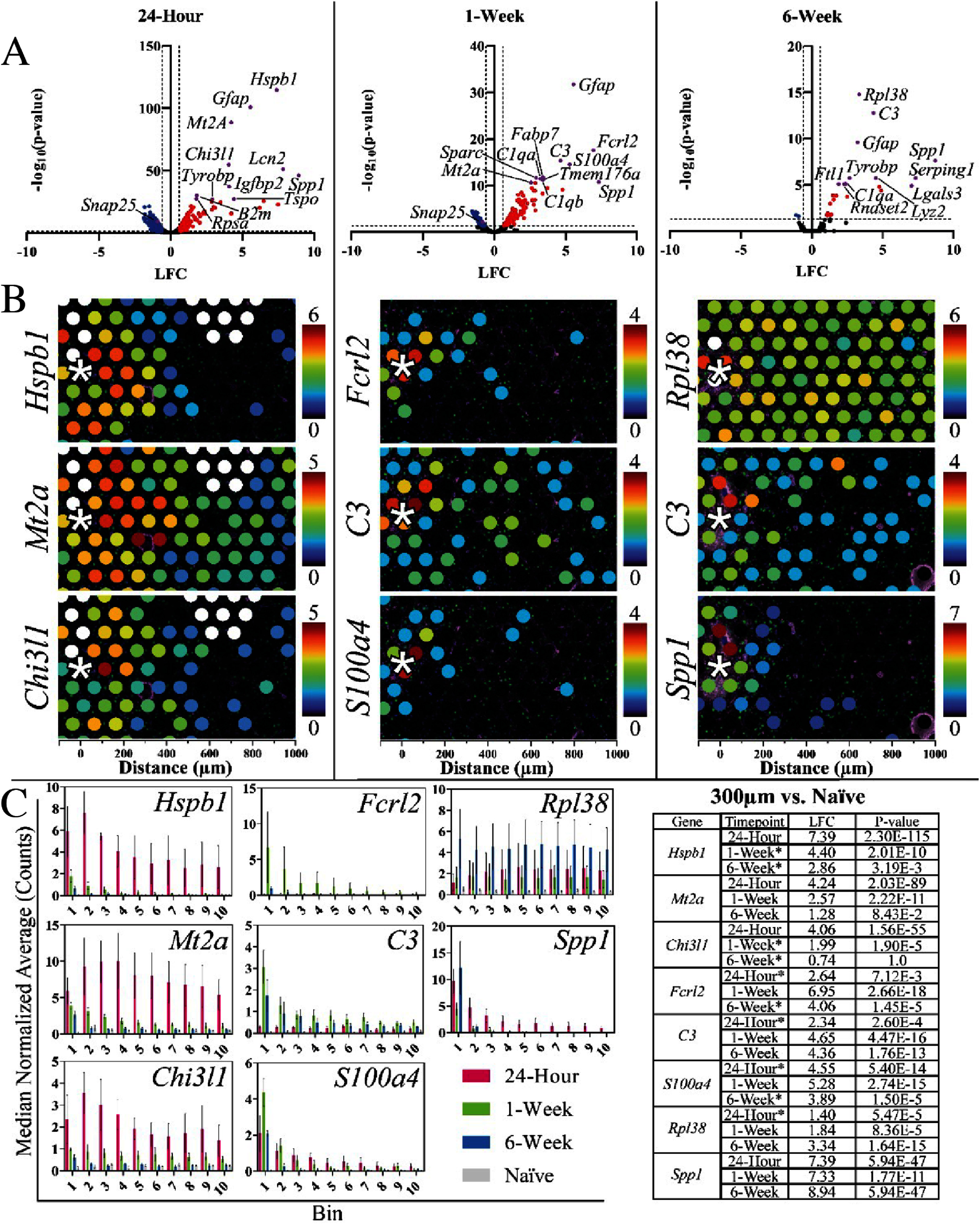
Differential expression and spatial expression of significantly expressed DE genes. (A) Volcano plots show the DE between a 300 *µ*m radius area surrounding the implant in 3 separate tissue samples at each timepoint compared to a 300 *µ*m radius area in similar locations of naïve, non-implanted tissue. (B) Images show the spatial gene expression of the 3 most significant DE genes in each of these timepoint comparisons, ordered by most to least significant, top to bottom. (C) Shows the bar graphs of MNA for each of the top 3 significant genes at each time point, and the table shows the LFC between the naïve and the other available time points for 300 *µ*m radii from the neural implant site.

To investigate overall changes in gene expression between implanted and naïve tissue, clusters of spots were compared to one another. Comparing spots with centroids within 300 *µ*m of the device tract to the same area of spots in naïve tissue sections revealed 553 DE genes at 24 h, 282 DE genes at 1 week, and 25 DE genes at 6 weeks post-implantation (figure 3). DEGs at 24 h were mostly downregulated compared to naïve tissue while DE genes at 1 week had roughly the same number of up and down regulated genes, and DE genes at 6 weeks were mostly upregulated. *Gfap* was one of the top three significant DE genes at all timepoints, while *Snap25* was significantly downregulated at 24 h and 1 week only.

The top 10 significant DE genes at each timepoint are shown in figure 3 and supplementary figures 15, 16. Panels in these figures show the spatial distribution of each gene’s expression in a representative image, the average expression in MNA counts at different ranges from the interface (bar graphs), and a table showing the LFC of each gene at each timepoint and its significance. Not including *Gfap* and in descending order of significance, the top 10 DE genes at 24 h post-implantation were Heat Shock Protein Family B (Small) Member 1 (*Hspb1*), Metallothionein 2A (*Mt2a*), Chitinase 3 Like 1 (*Chi3l1*), Lipocalin (*Lcn2*), Secreted Phosphoprotein 1 (*Spp1*), Insulin Like Growth Factor Binding Protein 2 (*Igfbp2*), Beta-2-Microglobulin (*B2m*), Ribosomal Protein SA (*Rpsa*), Translocator Protein (*Tspo*), and Transmembrane Immune Signaling Adaptor TYROBP (*Tyrobp*). At 1 week, the top 10 significant DE genes were Fc Receptor Like 2 (*Fcrl2*), Complement C3 (*C3*), S100 Calcium Binding Protein A4 (*S100a4*), Fatty Acid Binding Protein 7 (*Fabp7*), Secreted Protein Acidic and Cysteine Rich (*Sparc*), Complement C1q A Chain (*C1qa*), Transmembrane Protein 176A (*Tmem176a*), Complement C1q B Chain (*C1qb*), *Spp1*, and *Mt2a*. At 6 weeks, the top 10 significant DE genes were Ribosomal Protein L38 (*Rpl38*), *C3, Spp1, Tyrobp*, Lysozyme 2 (*Lyz2*), Serping Family G Member 1 (*Serping1*), *C1qa*, RibonucleaseT2 (*Rnaset2*), Ferritin Light Polypeptide 1 (*Ftl1*), and Galectin 3 (*Lgals3*). Full lists of DE genes may be found in supplemental data 1.

To examine physiological processes activated or suppressed by the significant DE genes at each timepoint, GO analysis was implemented using the Gene Ontology Enrichment Analysis and Visualization Tool (GOrilla). At 24 h post-implantation, a few significant GO Terms were ‘Positive Regulation of Glial Cell Proliferation’ (GO:0060252, FDR: 1.39 × 10^−2^), ‘Apoptotic Signalling Pathway’ (GO:0097190, FDR: 4.74 × 10^−2^), and ‘Response to Cytokine’ (GO:0034097, FDR: 6.76 × 10^−3^). At 1 week post-implantation, a few significant GO Terms were ‘Negative Regulation of Neuron Projection Development’ (GO:0010977, FDR: 6.69 × 10^−3^), ‘Complement Activation, Classical Pathway’ (GO:0006958, FDR: 1.87 × 10^−3^), and ‘Synapse Pruning’ (GO:0098883, FDR: 1.71 × 10^−3^). The 25 significant DE genes at 6 weeks post-implantation did not return any results from GO analysis, likely due to the small number of genes. A full list of significant GO Terms at the 24 h and 1 week timepoints are shown in supplementary data 2.

### 3.4. Computational analysis

Principal component analysis was performed on the MNA gene expression across the entire tissue and for ⩽300 *µ*m from the electrode site for every animal.

**Figure 4.**
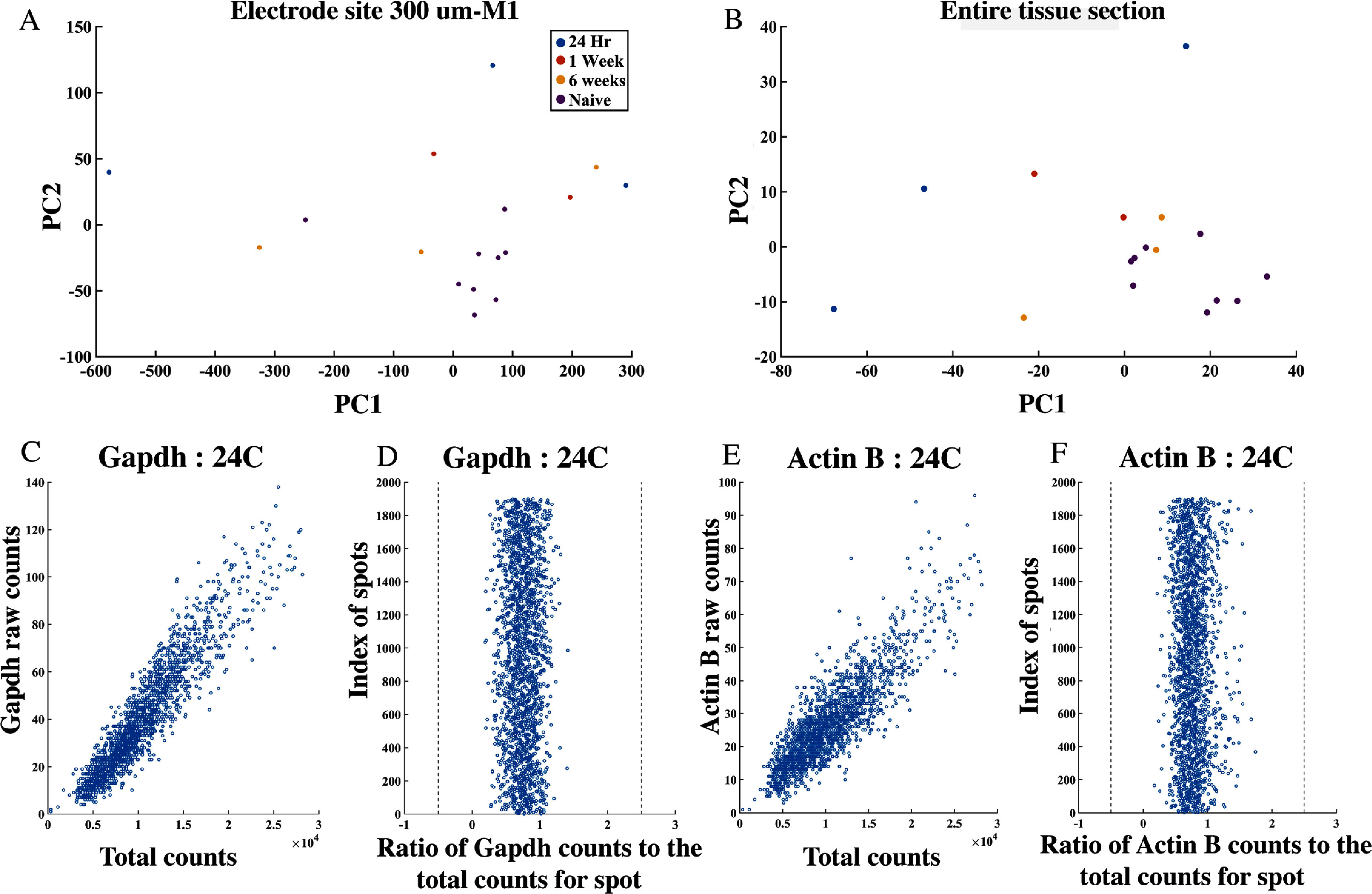
Initial inspection of data using principal component analysis (PCA) and analysis of housekeeping genes. (A) PCA of spots within M1 (<300 *µ*ms from device) indicate device-induced variability in gene expression in comparison to naïve samples. (B) Results from the whole tissue sample indicate the greatest variation in samples at the earliest time point post-injury (24 h). The later time points (1 and 6 weeks) display comparatively closer clustering, as well as overlap with unimplanted tissue in the PCA space, potentially indicating a partial resolution of device-induced gene expression effects over time. Selected example plots for two common housekeeping genes, *GAPDH* (D), (E) and *Actin-B* (F), (G), show the expected linear relationship between their normalised counts and the normalised total counts at each spot.

PCA of the MNA of local gene expression (⩽300 *µ*m from the electrode site) revealed clearer clustering in naïve tissues relative to implanted tissues (figure 4(A)), which displayed a higher degree of variability. It was observed that the 24 h animals have high variance between each other, which decreases for the 1 week and 6 week animals across the first two principal components (figure 4(B)). This is potentially explainable by sources of variation related to insertional damage and vascular disruption. This tissue damage and extravasation of blood, which inevitably occurs during the implantation process, triggers an immediate rush of inflammatory-mediating cells to the area [15, 56]. The later chronic phase of the tissue response is characterized by formation of a glial sheath around the electrode, in concert with neuronal loss.

This shift from insertional damage to a chronic tissue response might explain the tighter clustering of 1 week and 6 week time points compared to the highly variable 24 h time point (figure 4(B)), as well as the overlap of 6 week and naïve data in PCA space [15, 57, 58]. The counts of two well-known housekeeping genes were inspected with respect to the total counts, to check for any inconsistencies at any specific spot. The ratio of housekeeping gene counts with the total UMI counts at that spot should represent a linear relationship within an accepted range [35, 36].

Regression analysis was performed to identify the genes with the strongest predictive value of signal quality. PCR is the chosen method of regression for more than 1 genes, because there are relatively few observations (8 samples) compared to the number of independent variables (∼650 genes). Since PCR uses the principal components for regression it decreases the number of independent variables down to a smaller, more manageable number of uncorrelated variables [43]. Only the first component is used for regression of the MNA values of the electrode site, and the top genes of interest are genes that display both a highly positive or negative LFC (indicating a pronounced effect of device implantation) and a high *R* ^2^ value (indicating a strong predictive value for a recording quality or histological measurement of interest). We performed PCR on small groups of genes and found that genes associated with neuroinflammation and gliosis (e.g. *Lcn2, Hspb1, Lgals3*) were reliably associated with the output metrics of interest (supplementary figure 11).

**Figure 5.**
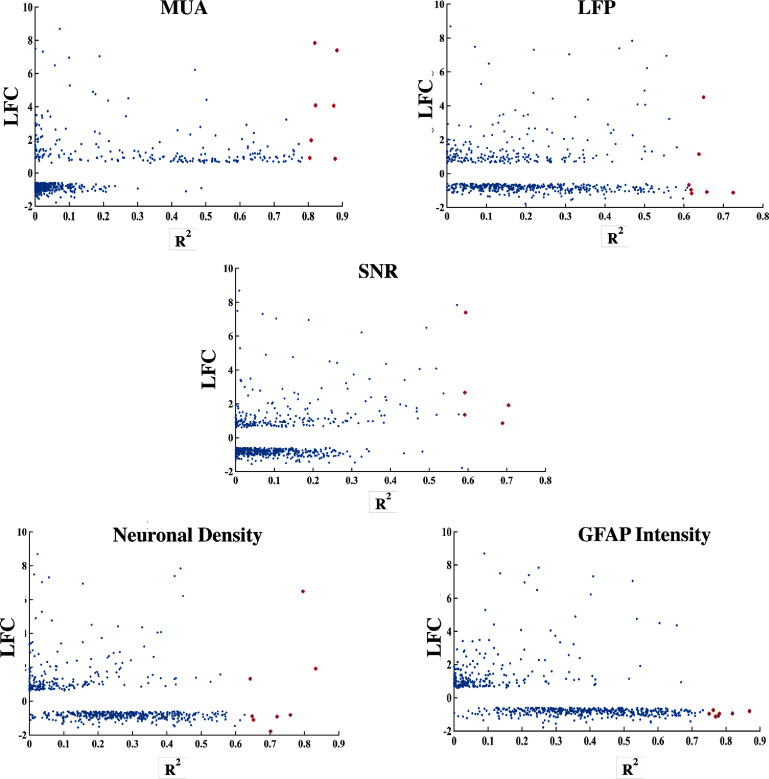
Regression and LFC were used to identify a subset of genes of interest. The 5 plots show where each gene lies on the axis of LFC and *R* ^2^ statistic, the genes shown in red are the genes that represent the highest value for both the parameters and were chosen for further investigation.

**Figure 6.**
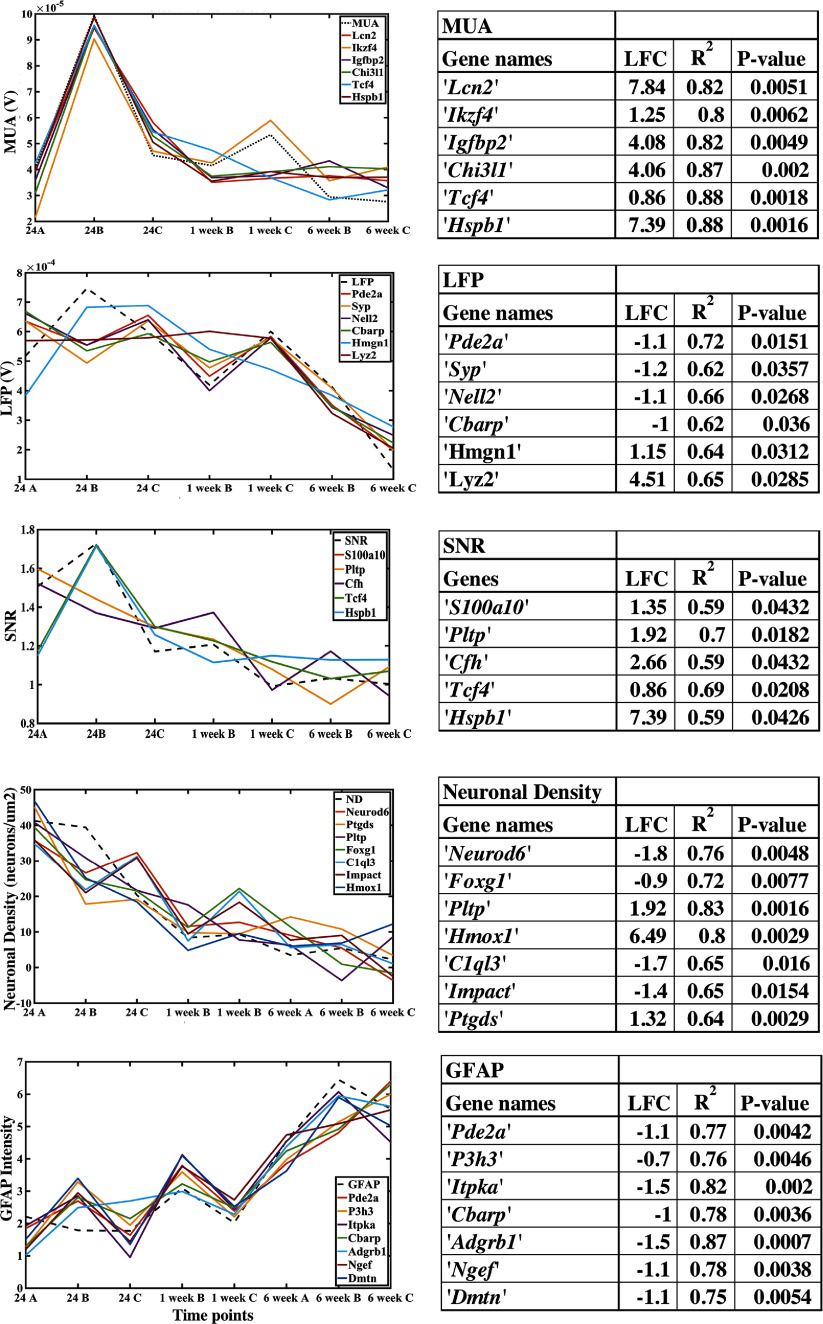
Applying the proposed pipeline identifies 6–7 genes of interest that are potential biomarkers for recording quality and histological metrics. (A) The tables show the genes selected through LFC and regression analysis for each observed variable: MUA (genes related to neuroinflammation, neuroprotection), LFP (synaptic functionality), SNR (neuroprotection, astrogliosis, neurodevelopment), ND (neuroprotection, astrogliosis) and GFAP (structural plasticity, synaptic and dendritic regulation). The plots compare estimated values of the observed variable predicted by the genes in the table through linear regression versus the actual values.

However, since individual genes are more easily targeted experimentally than groups of genes, we repeated the analysis for single genes (Scatter plot of LFC vs R2 value: figure 5, linear regression: figure 6). These results displayed similar genes and related signalling pathways, discussed below.

**Figure 7.**
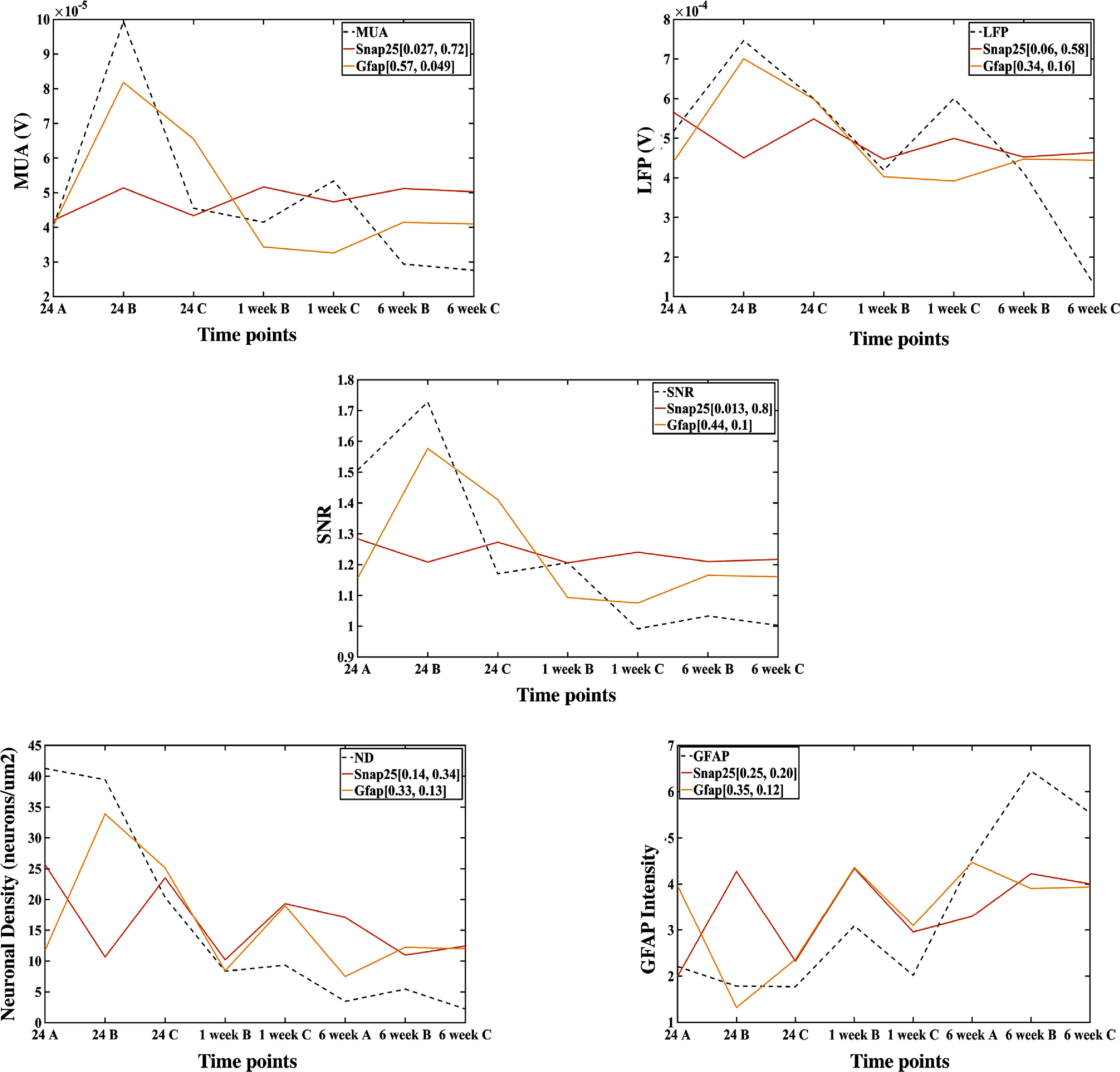
Cell type-specific marker genes, *Gfap* and *Snap25,* are poorer predictors of the histological and signal quality metrics than the genes predicted by regression. The table shows the *R* ^2^ and the non-adjusted *P*-values of the regression for both *Snap25* and *Gfap* in the brackets by the legend of each gene [*R* ^2^, non-adjusted *P*-value]. The *R* ^2^ values are low for both *Snap25* (∼10%–25%) and *Gfap* (∼35%–50%) compared to the genes identified through regression. The plots show the fit of the estimated values of the observed variables by *Gfap* and *Snap25* through regression, and it is visible that *Snap25* is a poor predictor of the respective observed variable than *Gfap.*

As a benchmark for comparison, we also assessed the *R* ^2^ statistic using PCR for *Snap25* and *Gfap* (figure 7), since these two genes are common markers for neurons and astrocytes and followed expected trends for neuronal density and astroglial activation (figures 1 and 2).

The *R* ^2^ statistic for *Gfap* is ∼ 50% for LFP, ND and GFAP, and close to 70% for MUA (only significant for MUA). The predictive quality of *Snap25* was poorer (non-significant for each metric). Thus, cell type-specific marker genes associated with traditional neuronal density and astroglial reactivity responses were poorer predictors of recording quality in comparison to genes revealed in the PCR analysis, which were more strongly associated with neuroinflammatory pathways and synaptic function.

## 4. Discussion

Recent reports in the literature highlight the value of applying transcriptomics techniques to understand the biological response to electrodes implanted in the brain. This report details an application of a ST assay that expands the current observations of the tissue response surrounding electrode implants in multiple ways. First, the ability to flexibly select ‘spots’ for differential expression analysis is a key advantage for understanding the spatial distribution of up- or down-regulated genes. Second, the combination of transcriptomics and IHC within the same tissue sample reveals new information about the source of genes of interest found in studies of the FBR to implanted electrodes: we can analyse the enrichment of gene expression relative to the distribution of specific cell types using IHC. An additional advantage of the approach lies in its spatial breadth, which allows for visualization of changes in gene expression far beyond the first few hundred microns of tissue that are typically assessed with immunohistochemistry. The data revealed effects of the device on gene expression at locations several millimeters distally to the implant location. For example, multiple genes of interest related to reactive astrogliosis such as *Gfap, C3, Lcn2*, and *Vim* were not only upregulated at the brain-electrode interface, but also in locations elsewhere in implanted tissue. At 24 h post-implantation, widespread reactive astroglial gene upregulation was observed, extending throughout the whole tissue section in some cases. In one 1 week sample, a spot of increased GFAP protein expression ∼1 mm away from the electrode tract exhibited increased reactive astroglial genes, and in 2/3 of the 1 week samples, the glia limitans also displayed increased expression of these genes. This finding shows that the transcriptomic effects of electrode implantation are not limited to the brain-electrode interface but can be found at distances from the electrode tract that are often not visible in images collected using traditional staining and microscopy techniques.

The observation of increased expression of genes associated with reactive astrogliosis in the glia limitans suggests that the transcriptomic effects of implantation may not only extend down the electrode tract, but also across the cortical surface and throughout the brain. What are the implications of this observation? The literature available to interpret this effect is relatively limited, with a notable exception of a recently published overview of astrocytic barriers^65^. It is possible that the glia limitans and device-associated glial encapsulation might be functioning in coordination, essentially amplifying the inflammatory response. While it is not yet clear how differential expression of inflammatory genes in these external areas affects the surrounding tissue, if the inflammation does affect the structure or function of the adjacent neural parenchyma, the effects of the FBR to implanted electrodes could extend distances across the surface of the cortex and throughout the brain orders of magnitude greater than previously reported. This could be an especially important consideration in the case of multiple implants.

In assessing the spatial expression pattern of the entire set of genes detected, a natural first analysis step is to generate lists of highly DE genes and explore those lists for relationships to known physiological processes. Similarly to previous studies, the top 10 genes that were DE in the region <300 *µ*ms from the implant site relative to naïve tissue included markers related to astrocyte reactivity (*Lcn2* [59, 60], *Chi3L1* [61, 62]), neurodegeneration(*Hspb1, Lcn2*) [59, 60, 63], intracerebral haemorrhage (*Mt2a* [64]), and neuroinflammation (*C1*, *C3*, *Spp1* [65, 66])). However, while analysing the whole transcriptome can reveal new and previously unreported potential biomarkers of device-tissue interaction, detailed discussions of these individual genes in isolation fails to identify those genes with a relationship with practical metrics of interest: namely, recorded signal quality.

To identify the individual genes with the strongest relationship with MUA and LFP amplitudes, as well as neuronal density and GFAP intensity, we performed regression analysis to identify the genes with the strongest predictive quality for each outcome measurement. Inspecting the top genes predictive for MUA amplitude reveals markers associated with both neuroinflammation and neuroprotection. For example, Lipocalin-2 (*Lcn2*) is induced by inflammation and can have both neuroprotective and neurodegenerative functions. *Lcn2* has been shown to act as an ‘help me’ signal produced by neurons which guides astrocytes and microglia into pro-recovery phenotypes [59]. However, under iron-mediated stress conditions or exposure to high concentrations for prolonged periods of time, *Lcn2* may increase cell death in neurons and astrocytes [59, 60]. *Ikzf4* together with *Ikzf1* have been identified as neuronal reprogramming factors, having been shown to reprogram fibroblasts into induced neurons and convert uninjured retinal glia into neuron-like cells [67, 68]. The glycoprotein *Chi3l1* is predominantly expressed by astrocytes. Microglia-induced *Chi3l1* secretion from astrocytes augments inflammation and promotes neural damage [59]. Interestingly, *Chi3l1* has been associated with suppression of glial phagocytic activation [62]. *Hspb1* is highly reactive to oxidative stress, neuroinflammation and has a regulating effect on acute neuroinflammation by intensifying the expression of pro-inflammatory cytokines and enhancing glial cell activation, but not increasing neuronal apoptosis [69, 70]. Generally, the genes that were most strongly predictive of MUA amplitude were related to neuroinflammation; additionally, *Igfbp2* and *Tcf4* are related to neuritogenesis and dendritic structure, respectively [71–73].

Synapse-associated genes dominate the list of top predictors of LFP amplitude, in accordance with the interpretation that the LFP is predominantly generated by synaptic conductances [33]. *Pde2a* belongs to a family of enzymes involved in the homeostasis of both cAMP and cGMP [74]. In the brain, both cAMP and cGMP are essential during neurodevelopment as well as in maintaining synaptic plasticity, and they have critical roles in axon elongation and guidance [75, 76]. In turn, cAMP abundance coupled to PKA signalling is critical to modulate assembly/disassembly/priming/recycling of neurotransmitter vesicles and, consequently, for synaptic transmission and plasticity events. Synaptophysin is an integral membrane protein localized to synaptic vesicles. *Syp* is the most abundant protein of synaptic vesicles by mass [77]. Neural epidermal growth factor-like like 2 (*Nell2*) is a cytoplasmic and secreted glycosylated protein with six epidermal growth factor-like domains. *Nell2* is predominantly expressed in neural tissues where it regulates neuronal differentiation, polarization, and axon guidance [78]. A detailed analysis of expression during oligodendrogenesis revealed that the majority of oligodendroglia express *Nell2* [79]. *Nell2* promotes survival of neurons by modulating MAPK activity [79].

Signal quality is influenced by a variety of factors, both biological and non-biological. Reactive glia may serve as a physical barrier to signal transmission or influence neuronal signals through release of cytokines [15]. The neurons may be dysfunctional, despite being observable with NeuN labeling (e.g., neurons may have structural damage to dendritic arbors, spine loss, or alterations in electrophysiological responses) [52]. SNR perhaps captures these factors in a more wholistic fashion, being reflective of sources of both signal and noise. Genes associated with SNR can be categorized as having functions in neuroprotection (*s100a10* [80, 81], *Pltp* [82, 83]), dendritic structure (*Tcf4* [72, 73]), cortical development (*Tcf4, Cf*h) [73, 84] and oxidative stress/neuroinflammation (*Hspb1)* [69, 70]. *S100a10* has been identified as a marker of neuroprotective astrocytes [80]. It has also been shown to be an essential negative regulator of toll like receptor (TLR) function and a potential therapeutic target for treating inflammatory diseases [81]. TLR signalling influences multiple dynamic processes in the developing and adult CNS including neurogenesis, axonal growth, and structural plasticity.

While we were primarily interested in identifying genes predictive of recording quality, we also assessed genes that were strongly associated with traditional markers of the tissue response to implanted electrodes. Genes strongly associated with neuronal density have known relationships to neuroprotection (*Hmox1*) [85], astrogliosis, and astrocyte-mediated neuroprotection (*Pltp*) [82, 83]. The gene lists generally supports the hypothesis that neural proliferation, differentiation, and neuritogenesis is inhibited surrounding the implant and that shortening of dendritic branches and neuronal death is promoted in the vicinity of the neural prosthesis. For the genes associated with GFAP staining intensity, functions included synaptic physiology (*Itpka*) [86], structural plasticity (*Adgrb1*) [87], angiogenesis, regulation of dendritic spine and excitatory synapse development, and neuroprotection [88, 89].

Notably, the genes that were most strongly associated with signal quality were distinguished from those with the strongest association with the tissue response. Likewise, *Snap25* and *GFAP*, typical markers for neurons and astrocytes, were relatively poorer predictors of recording performance. An interpretation of these phenomena is that the biological determinants of performance are incompletely captured by neuronal density and GFAP intensity. The genes predictive of signal quality in this study should be explored further to confirm their generalizability as biomarkers of signal quality: many experimental factors could influence results. It is important to contextualize the results of our analysis with several considerations, including: (1) our recording metrics may not capture all aspects of quality relevant to the variety of device applications in use, (2) relatively low sample size (although, our sample sizes are aligned with reports in literature using ST assays [21, 47–49]) and (3) the potential to over-interpret causation when assessing the results of regression. Additionally, it will be important to include additional time points in future work to assess the transition between acute and chronic-phase response, as well as gene expression beyond the 6 week timeframe. It will be important to assess relationships for individual time points and phases of the tissue response (insertional damage versus subsequent acute and chronic phase responses). We would like to emphasize that the goal of this paper regarding the computational pipeline used is not to establish causality between the dependent variables and the genes. Our goal was to provide a preliminary exploration of the relationship between the genes and dependent variables in order to generate hypotheses for investigation. Further work is required. Nonetheless, we have developed a new approach to understand the tissue response to electrodes implanted in the brain using genes identified through transcriptomics, and to screen those results for potential relationships with functional outcomes. Our analysis has shed new light onto the potential mechanisms involved in the tissue response to implanted electrodes while generating hypotheses regarding potential biomarkers related to recorded signal quality. Future work will need to directly test the causative nature of these markers through pharmacological or genetic manipulation.

## Data Availability

The data that support the findings of this study are openly available at the following URL/DOI: www.ncbi.nlm.nih.gov/sra/PRJNA1089183.
